# The emerging roles of non-canonical ubiquitination in proteostasis and beyond

**DOI:** 10.1083/jcb.202311171

**Published:** 2024-03-22

**Authors:** Yoshino Akizuki, Stephanie Kaypee, Fumiaki Ohtake, Fumiyo Ikeda

**Affiliations:** 1 Institute for Advanced Life Sciences, Hoshi University, Tokyo, Japan; 2 Graduate School of Frontier Biosciences, Osaka University, Osaka, Japan

## Abstract

Ubiquitin regulates various cellular functions by posttranslationally modifying substrates with diverse ubiquitin codes. Recent discoveries of new ubiquitin chain topologies, types of bonds, and non-protein substrates have substantially expanded the complexity of the ubiquitin code. Here, we describe the ubiquitin system covering the basic principles and recent discoveries related to mechanisms, technologies, and biological importance.

## Teaser

Akizuki et al. review our latest knowledge of the roles of non-canonical ubiquitination in protein stability and signal transduction.

## Introduction

The ubiquitin–proteasome system is an important anticancer target, with proteasome inhibitors showing efficacy as cancer therapeutics (Goldberg, 2012). In addition, targeted protein degradation via the ubiquitin–proteasome system has emerged as a transformative new modality in drug discovery (Burslem and Crews, 2020). Ubiquitin modifications also serve to modulate protein–protein interactions and signaling pathways (Komander and Rape, 2012). By forming different linkage types of ubiquitin chains on target substrates, ubiquitin regulates various cellular functions. In this review, we summarize recent insights into the functions of canonical and non-canonical ubiquitination in the regulation of cellular signaling as well as targeted protein degradation.

### Ubiquitination

**Figure 1.**
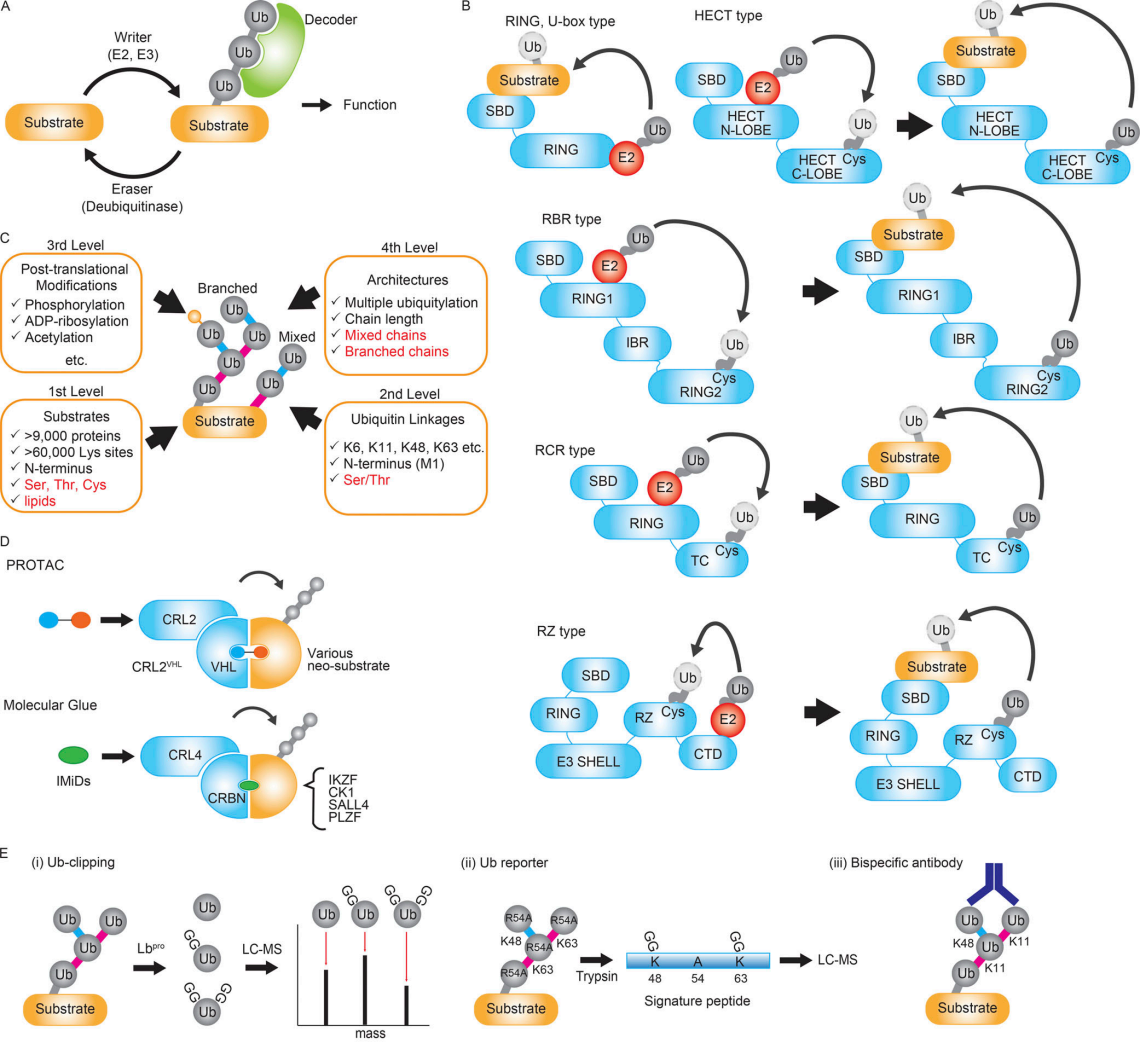
**The basics of ubiquitination. (A)** The ubiquitin code is generated by the E1, E2, and E3 enzymes (the writers), disassembled by the deubiquitinases (the erasers), and translated by the ubiquitin-binding domain-containing proteins (the decoders). **(B)** The mechanism of ubiquitin transfer from the E2 enzyme to a substrate depends on the type of E3 enzyme. E3 ligases include the RING and U-box type, the HECT type, the RBR type, the RCR type, and the RZ type. SBD: substrate binding domain. **(C)** The ubiquitin code has four levels of complexity: type of substrates, ubiquitin chain linkage type, posttranslational modification of ubiquitin, and the architecture of ubiquitin chains. Recently discovered ubiquitin codes are highlighted in red and described in this review. **(D)** Targeted protein degradation. Schemes for the action of PROTAC-type and a molecular glue–type degraders are shown. **(E)** Representative methodologies to quantify branched ubiquitin linkages. (i) In Ub-lipping, ubiquitin moieties generated by the enzyme Lb^pro^ are analyzed by mass spectrometry to distinguish branched linkages. (ii) The K48/K63 branched linkages can be quantified by expressing ubiquitin R54A mutant as a reporter. (iii) The K11/K48 bispecific antibody can recognize K11/K48 mixed and branched ubiquitin chains.

Ubiquitin is a 76-amino acid (aa) protein that is conserved in eukaryotes and regulates diverse processes, generally as the source of a posttranslational protein modification (Oh et al., 2018). Ubiquitination proceeds through three sequential reactions catalyzed by ubiquitin-activating (E1), conjugating (E2), and ligating (E3) enzymes. The first step, ubiquitin activation, is ATP-dependent and links ubiquitin to an E1. An E2 replaces the E1 during the conjugation step, and an E3 transfers the ubiquitin from the E2 to the target substrate during the final ligation step. E3 ubiquitin ligases (∼600 proteins in humans) recognize specific substrates, thus providing substrate specificity in the ubiquitin system (Fig. 1 A).

Among the six known E3 families, the “Really Interesting New Gene” (RING) type and the “U-box” type E3s simultaneously interact with both the substrate and E2 to facilitate ubiquitin transfer (Fig. 1 B). In contrast, the “homologous to E6AP C terminus” (HECT), “RING-in between-RING” (RBR), “RING-Cys-relay” (RCR), and RZ type E3s conjugate ubiquitin to themselves via a thioester bond and then directly transfer the ubiquitin to the substrate. The ubiquitin code is interpreted by decoders (Komander and Rape, 2012) and eventually erased by specific deubiquitinases (DUBs) (Clague et al., 2019) (Fig. 1 A).

### The ubiquitin code: Multiple levels of complexity

Conjugated ubiquitin can be further modified, for instance, by the addition of another ubiquitin to assemble a polyubiquitin chain. Ubiquitin chains can be linked through one of their seven lysine (K) residues or the first methionine (M1), resulting in at least eight types of polyubiquitin chains (Komander and Rape, 2012). Distinct ubiquitin chains establish a “ubiquitin code” that serves diverse functions. Recent advances have uncovered the high complexity ubiquitin code (Dikic and Schulman, 2022; Ikeda, 2023; Kelsall, 2022; Sakamaki and Mizushima, 2023; Squair and Virdee, 2022). To capture these complexities, we describe four levels (Fig. 1 C).

The first level of complexity corresponds to substrate diversity. This level includes >9,000 known substrate proteins with >60,000 lysine sites, as revealed by proteomics analyses (Rose et al., 2016). The specific ubiquitination sites within a substrate impact the outcome, in part because ubiquitination can affect the structure of the substrate, such as promoting the formation of disordered regions that accelerate proteasomal degradation (Carroll et al., 2020). In addition to lysine, other residues such as serine (S), threonine (T), cysteine (C), and the N-terminus M1 can also be ubiquitinated (Ikeda, 2023; Kelsall, 2022; Squair and Virdee, 2022). Ubiquitin was also recently shown to modify non-protein substrates, such as lipids and sugars (Otten et al., 2021; Sakamaki and Mizushima, 2023; Sakamaki et al., 2022).

The second level of complexity corresponds to the diverse types of linkages that create polyubiquitin chains. Polyubiquitin chains can be linked through their K residues, the first M (M1) residue, and through S and T residues. K48-linked are considered the canonical ubiquitin chain, and these act as a proteasome-targeting signal to mediate substrate degradation (Hershko and Ciechanover, 1998). In contrast, K63- or M1-linked chains act as non-degradation tags regulating inflammatory signaling pathways (Komander and Rape, 2012; Swatek and Komander, 2016). S/T-linked ubiquitination has important cellular roles in inflammatory responses and aggregate formation (Kelsall et al., 2022; Petrova et al., 2021; Rodriguez Carvajal et al., 2021).

The third level of complexity reflects other posttranslational modifications of ubiquitin itself (Fig. 1 C) (Swatek and Komander, 2016). These modifications include acetylation, ADP-ribosylation, and phosphorylation (Dikic and Schulman, 2022). For instance, during Parkin-mediated mitophagy, the kinase PINK1 and E3 ligase PARKIN are recruited to damaged mitochondria, where PINK1-dependent phosphorylation of ubiquitin and PARKIN are induced (Herhaus and Dikic, 2015). These phosphorylation events are critical for mitochondrial degradation by mitophagy (Gladkova et al., 2018; Gundogdu et al., 2021; Sauve et al., 2018).

The fourth level of complexity, “architecture,” relates to the higher-order structures of ubiquitin chains determined by the chain length and the linkage topology (Fig. 1 C). The length of the ubiquitin chain impacts function. For instance, the proteasome was proposed to recognize ubiquitin that are tetramers or longer (Thrower et al., 2000), and structural analysis revealed that the p97/Valosin-containing protein (VCP)-NPLOC4 (NPL4)- ubiquitin fusion degradation 1 (UFD1) complex recognizes K48-linked pentamer chains (Twomey et al., 2019). The original concept that ubiquitin chain length is the key determinant of proteasomal degradation has been modified that there are diverse ubiquitin signals that promote proteasomal degradation; multiple oligo-ubiquitination also serves as a proteasomal signal (Lu et al., 2015). Moreover, the “branched” ubiquitin chains have been proposed as a priority signal for proteasomal degradation (Kolla et al., 2022). Given the multiple conjugation sites within a single ubiquitin molecule, chains can comprise different linkages in tandem (a mixed chain, Fig. 1 C), or two parallel ubiquitin moieties with distinct linkages to one ubiquitin called a branched ubiquitin chain (a branched chain, Fig. 1 C). Mixed and branched chains have recently emerged as versatile signals that can amplify, inhibit, or convert the information of a parental, homotypic ubiquitin code (French et al., 2021; Haakonsen and Rape, 2019; Kolla et al., 2022).

Based on the recent advancement, we focus on three particular areas of ubiquitin substrates, ubiquitin linkages, ubiquitin architectures as highlighted in red in Fig. 1 C.

### Targeted protein degradation

Targeted protein degradation is attracting attention not only as a groundbreaking therapeutic strategy but also as a new research tool for cell biology (Verma et al., 2020). PROTACs (PROteolysis TArgeting Chimeras) are molecules that bridge artificially targeted proteins (neosubstrates) to E3 ubiquitin ligases, inducing ubiquitination and proteosome-mediated degradation of the neosubstrates (Burslem and Crews, 2020; Gadd et al., 2017; Verma et al., 2020) (Fig. 1 D). Notably, a PROTAC that targets estrogen receptor alpha (ER-α) recently entered clinical trials with patients with breast cancer (Chirnomas et al., 2023). The E3 ligases VHL (a component of CRL2VHL ligase) and cereblon (CRBN, a component of CRL4CRBN) are often used for targeted protein degradation owing to their efficiency in ubiquitinating and degrading neosubstrates (Samarasinghe and Crews, 2021; Verma et al., 2020).

The formation of a stable neosubstrate–PROTAC–E3 ternary complex is critical for degradation (Gadd et al., 2017). In addition, the K48-specific E2s UBE2G and UBE2R are required for the degradation of neosubstrates, suggesting that this process depends on the formation of K48-linked ubiquitin chains (Mayor-Ruiz et al., 2019; Hill et al., 2019; Lu et al., 2018; Sievers et al., 2018). Regulators of the cullin neddylation cycle, such as the COP9 signalosome (CSN) (Mayor-Ruiz et al., 2019), and the ubiquitin-dependent segregase/unfoldase p97/VCP (Nguyen et al., 2017) are also involved in the targeted degradation of neosubstrates. Given that the strategy depends on ectopic ubiquitination, DUBs might counteract neosubstrate degradation. Indeed, the DUB USP15 has been reported to antagonize CRL4CRBN-mediated ubiquitination of neosubstrates (Nguyen, 2021).

Molecular glues are another class of chemical degraders, similar to PROTACs, and also bring neosubstrates into the proximity of E3s (Jan et al., 2021) (Fig. 1 D). The immunomodulatory drugs thalidomide and its derivatives (e.g., lenalidomide and pomalidomide), are prototypical molecular glues used as therapeutics for multiple myelomas (Jan et al., 2021). These drugs were found to be cytotoxic only in multiple myeloma cells expressing CRBN (Lopez-Girona et al., 2012; Zhu et al., 2011), and it was subsequently discovered that they act by targeting essential β-hairpin-containing neosubstrates for CRBN-dependent proteasomal degradation (Jan et al., 2021). VCP/p97 is required for the thalidomide-induced degradation of the neosubstrates IKAROS Family Zinc Finger (IKZF)1/3 (Nguyen et al., 2017).

### Functions and assembly of branched ubiquitin chains

There are many different kinds of branched ubiquitin chains, and they are involved in myriad cellular processes. For instance, the well-studied K11/K48-branched ubiquitin chains are potent signals for proteasomal degradation of mitosis-related regulators (Meyer and Rape, 2014), and also play critical roles in the quality control of aggregate-prone proteins, such as Huntingtin (Yau et al., 2017). K48/K63-branched ubiquitin chains are another abundant branched linkage in mammalian cells and yeast (Ohtake et al., 2016; Pluska et al., 2021). During mitophagy, the RBR-type E3 Parkin monoubiquitinates the substrate and also assembles short-branched ubiquitin chains on mitochondrial membrane proteins (Swatek et al., 2019). Nuclear factor κB (NF-κB) inflammatory signaling is regulated by M1/K63 mixed ubiquitin chains, and K48/K63 branching of the mixed ubiquitin chains can inhibit their deubiquitination (Emmerich et al., 2013; Ohtake et al., 2016). Thus, diverse cellular pathways are regulated by the branched and mixed ubiquitin chains and by their responsible E2s/E3s (Gregor et al., 2023).

The mechanism of how the branched ubiquitin codes are recognized by the decoder proteins is still enigmatic. However, accumulating evidence suggests a role of branched chains in proteasomal degradation. In cell-based analyses, K11/K48, K48/K63, or K11/K48/K63 branched ubiquitin chains associate more efficiently with the proteasome (Akizuki et al., 2023; Meyer and Rape, 2014; Ohtake et al., 2018; Yau et al., 2017). K11/K48 branched chains associate with p97 (Yau et al., 2017), while K29/K48 branched chains associate with the proteasome as well as shuttling factor Rad23 and Dsk2 in yeast (Liu et al., 2017). More specifically, the K11/K48 branched trimer is recognized more strongly by the proteasomal subunit RPN1 in vitro (Boughton et al., 2020).

Certain methods have been applied to quantify the branched linkages. In Ub-clipping, an engineered viral protease Lbpro specifically cleaves ubiquitin after Arg74 (Swatek et al., 2019). The resultant ubiquitin moieties containing Gly75–Gly76 conjugated to target lysines are analyzed by a method called middle-down mass spectrometry to distinguish the ubiquitin moieties of branching points (Fig. 1 E [i]). Using the strategy, they found that around 10–20% of ubiquitin exist in the form of branched chains (Swatek et al., 2019). As for the K48/K63 branched linkages, a reporter ubiquitin harboring an Arg54 to Ala mutation was expressed in cells to quantify specific signature peptides using mass spectrometry (Fig. 1 E [ii]). It revealed that 20% of total K63 linkages are branched at K48 in cells and that the stoichiometry arises to 50% under proteasomal inhibition (Ohtake et al., 2016). Moreover, a bispecific antibody recognizing K11/K48 mixed ubiquitin chains (Fig. 1 E [iii]) revealed that the abundance of the K11/K48 mixed and branched chains increases at mitosis (Yau et al., 2017).

### Assembly of branched ubiquitin chains

**Figure 2.**
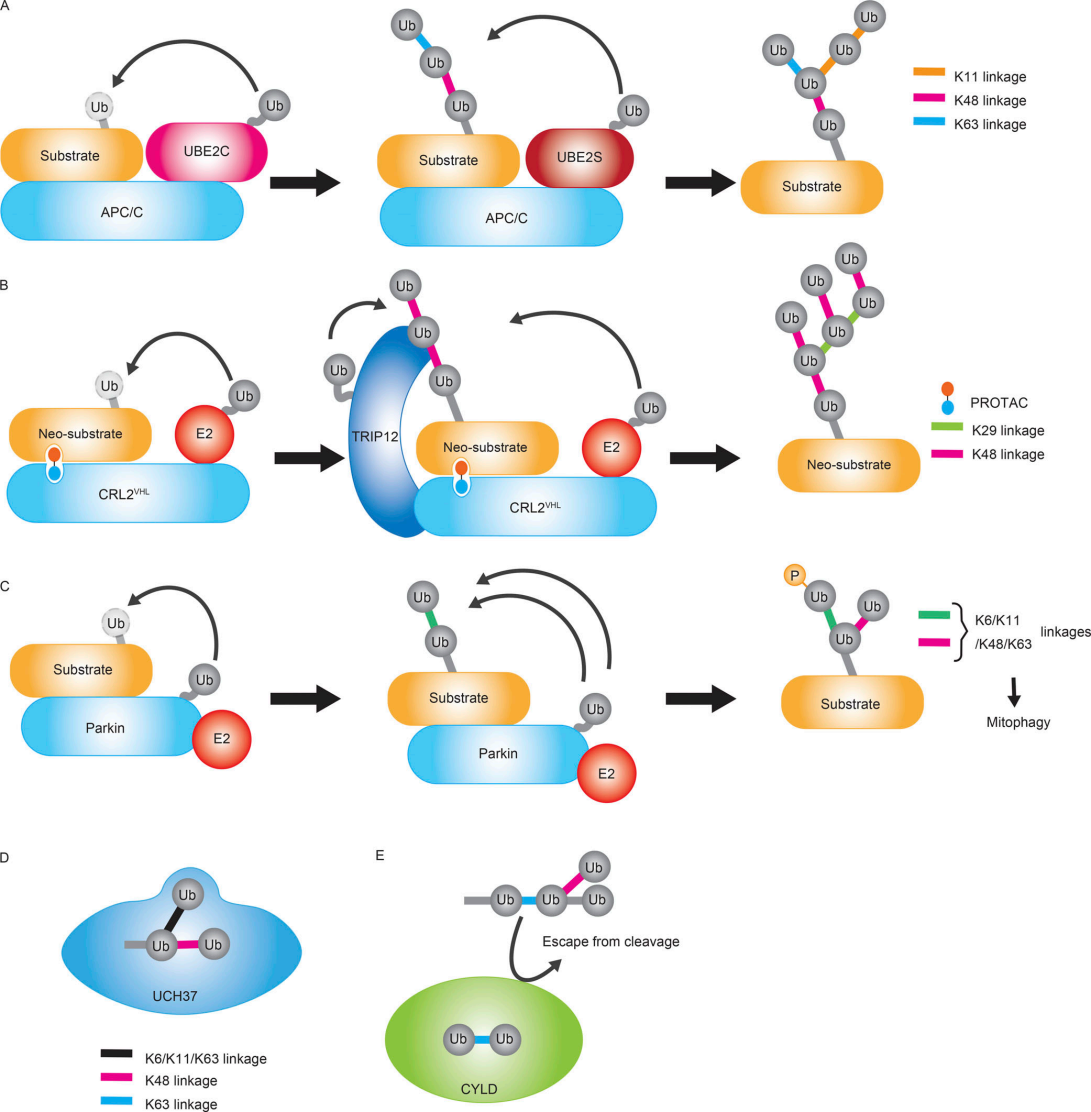
**Types of enzymes regulating branched ubiquitin chains. (A)** A single E3 assembles branched chains by recruiting two or more different E2s. APC/C recruits UBE2C to initiate short K11, K48, and K63 linked chains on the substrate. The subsequent association of UBE2S with APC/C results in the K11-linked branching on the pre-formed chains. **(B)** Two E3s cooperatively assemble branched chains. TRIP12 and CRL2^VHL^ with different linkage specificities cooperate to assemble K29/K48-branched ubiquitin chains on a neosubstrate targeted by a PROTAC, leading to proteasomal degradation. **(C)** A single E3 such as PARKIN can assemble multiple linkages including K6, K11, K48, and K63, resulting in the formation of the branched linkages. Ubiquitin can be also phosphorylated during mitophagy regulation. **(D)** The deubiquitinase UCH37 specifically cleaves K48 linkages within branched ubiquitin linkages. **(E)** The presence of K48 branches on the distal ubiquitin moiety of the K63-linked chains inhibits K63 chain disassembly by CYLD.

Branched ubiquitin chains can be synthesized in several ways (Fig. 2, A–C and Table 1). For instance, a single E3 can scaffold multiple E2s that work together to form a distinct branched ubiquitin chain. The E3 ligase anaphase-promoting complex (APC/C) acts in this way, binding to the E2 enzymes UBE2C and UBE2S to synthesize branched ubiquitin chains (Meyer and Rape, 2014) (Fig. 2 A). UBE2C initiates short chains of K11, K48, and K63 linkages, and UBE2S branches K11-linked ubiquitin chain off the previously formed chains (Meyer and Rape, 2014; Wickliffe et al., 2011; Williamson et al., 2011).

**Table 1.** E2s, E3s, and DUBs involved in the assembly or disassembly of branched ubiquitin chains

**Branching E3s**						
Linkage type	Mechanism	E2s and E3s	Substrates	Function	Technology	References	
K11/K48	A	APC/C + UBE2C +UBE2S	Cyclin A	Proteasomal degradation	Ub mutation	Meyer and Rape (2014)	
			NEK2A		Bispecific antibody	Yau et al. (2017)	
			Histone H2B		Bispecific antibody	Oh et al. (2020)	
	B	UBR5 + K11-specific E2/E3	73Q-HTT	Proteasomal degradation	Bispecific antibody	Yau et al. (2017)	
K29/K48	B	Ufd4 + Ufd2	Ub-V-GFP	Proteasomal degradation	Ub mutation	Liu et al. (2017)	
	B	CRL2^VHL^ or CRL4^CRBN^ + TRIP12	BRD4	Targeted protein degradation	Mass spectrometry, Ub-Clipping	Kaiho-Soma et al. (2021)	
	C	HECTD1	N/A	N/A	UbiCRest, Mass spectrometry	Harris et al. (2021)	
	C	UBE3C	N/A VPS34	N/A Proteasomal degradation	Ub mutation, Mass spectrometry	Wang et al. (2006)	
					Ub mutation	Chen et al. (2021)	
K6/K48	C	IpaH9.8	N/A	N/A	Mass spectrometry	Valkevich et al. (2014)	
	C	Parkin	N/A	Mitophagy	Ub-clipping	Swatek et al. (2019)	
	C	NlEL	N/A	N/A	UbiCRest	Hospenthal et al. (2013)	
			N/A	N/A	Mass spectrometry	Valkevich et al. (2014)	
K48/K63	B	ITCH + UBR5	TXNIP	Proteasomal degradation	Ub mutation, Mass spectrometry	Ohtake et al. (2018)	
	B	TRAF6 + HUWE1	TRAF6	NFkB signal activation	Mass spectrometry, Ub mutation	Ohtake et al. (2016)	
	A?	Ubc1/UBE2K	N/A	N/A	Ub mutation	Pluska et al. (2021)	
K11/K48/K63	C	WWP1	WBP2	N/A	Ub mutation	French et al. (2017)	
	A	cIAP1 + UBE2D + UBE2N/UBE2V	cIAP1, ER-α	Targeted protein degradation	Mass spectrometry, UbiCRest	Akizuki et al. (2023)	
K63/M1	B	TRAF6 + LUBAC	IRAK1	NFkB signal activation	UbiCRest	Emmerich et al. (2013)	
			RIP1 and RIP2	NFkB signal activation	UbiCRest	Emmerich et al. (2016)	
Multiple types	B	UBE2D + multiple RING E3s	Luciferase, troponin I	Inhibition of proteasomal degradation	Ub mutation	Kim et al. (2007)	

**DUBs**							
**Target branched linkage**	**Effect of branched chains**	**DUB**	**Family**	**Linkage specificity**	**Substrate**	**Function**	**References**
K29/K48	Retain	TRABID/ZRANB1	OTU	K29,K33>>K63	HECTD1	Protect from degradation	Harris et al. (2021)
K11/K48, K11/K63, M1/Oxyester		Cezanne	OTU	K11, Oxyester	APC/C substrates	Protect from degradation	Mevissen et al. (2016)
M1/Oxyester					N/A	Inhibit NF-kB signal	Rodriguez Carvajal et al. (2021)
K6/K48		UPS30	USP	K6	Parkin	Inhibit mitophagy	Deol et al. (2020b)
K63/M1	Inhibition	A20	OTU	K63	RIPK-1	Inhibit NF-kB signal	Wertz et al. (2015)
K63/K48		CYLD	USP	K63, M1	TRAF6	Inhibit NF-kB signal	Ohtake et al. (2016)
K6/K48, K48/K63	Promotion	UCH37	UCH	K48	N/A	Promote proteasomal degradation	Deol et al. (2020b)

Similarly, the E3 ligase cellular inhibitor of apoptosis protein 1 (cIAP1) scaffolds multiple E2s to assemble branched ubiquitin chains on itself during ligand-induced self-degradation (Akizuki et al., 2023). Activated cIAP1 is monoubiquitinated by the E2 enzyme UBE2D and then binds UBE2N/UBE2V, which forms K63-linked ubiquitin chains. Finally, UBE2D branches K11 and K48 linkages, resulting in K63 linkages proximal to cIAP1 and K11/K48 and K48/K63 linkages at the distal end of the ubiquitin chain. cIAP1 antagonists are in clinical trials for various cancers such as lymphomas and solid tumors (Cong et al., 2019; Morrish et al., 2020).

Alternatively, multiple E3s can collaborate to build branched ubiquitin chains. For instance, the HECT-type E3 ubiquitin-protein ligase E3 component N-recognin 5 (UBR5) partners with other E3s to synthesize branched K48 linkages off pre-existing ubiquitin chains. Structural analysis showed that oligomeric UBR5 specifically assembles K48 linkages by binding to ubiquitin chains with its ubiquitin-associated (UBA) domain (Hehl et al., 2023). UBR5 and UBR4 synthesize K11/K48 branched ubiquitin chains onto the polyglutamine repeat-containing protein 73Q-huntingtin (HTT) and facilitate its rapid degradation (Yau et al., 2017). Another aggregation-prone protein C9orf7 is a reported substrate of UBR5 and modified with a K11/K48 linkage controlling proteasomal degradation of C9orf72 (Julg et al., 2023). UBR5 also partners with the itchy E3 ubiquitin protein ligase (ITCH) and synthesizes K48/K63-branched ubiquitin chains on thioredoxin interacting protein (TXNIP) (Ohtake et al., 2018). ITCH directly interacts with TXNIP and modifies it with K63-linked chains, and UBR5 recognizes the K63-linked chains. K63-linked chains of TXNIP are required but not sufficient for its proteasomal degradation.

Intriguingly, the HECT-type E3 ligase 220 12 (TRIP12) enhances PROTAC-induced, CRL2VHL-dependent degradation of the neosubstrate BRD4 (Fig. 2 B). Mechanistically, Cullin 2 (CUL2) within CRL2VHL directly interacts with and recruits TRIP12 to BRD4, leading to TRIP12-dependent formation of K29 linkages on the K48-linked chains on BRD4. Ultimately, TRIP12 and CRL2VHL cooperatively assemble K29/K48-branched ubiquitin chains to promote neosubstrate degradation (Kaiho-Soma et al., 2021) (Fig. 2 B). Thus, the K29/K48 branched ubiquitin chain can be established by distinct E3–ubiquitin and E3–E3 interactions (Fig. 2 B). A yeast ortholog of TRIP12, UFD4, generates K29 linkages that serve as substrates for UFD2-mediated K48-linkage elongation (Liu et al., 2017). This specific type of branching suggests that K48 residues within K29-linked ubiquitin chains serve as good substrates for further ubiquitination.

The “HECT, UBA, and WWE domain-containing protein 1” (HUWE1), another E3 ubiquitin ligase, produces K48 branches on K63 chains generated by the E3 ligase TNF Receptor Associated Factor 6 (TRAF6), resulting in branched K48/K63 chains. This specific branched linkage safeguards K63 linkages from deubiquitination, while retaining their recognition by adapter protein TAB2, thereby enhancing NF-κB signaling (Ohtake et al., 2016).

The linear ubiquitin chain assembly complex (LUBAC) contains two E3 ligases, the RanBP-type and C3HC4-type zinc finger-containing protein 1 (HOIL-1)/RBCK1 and the HOIL-1-interacting protein (HOIP)/RNF31, as well as a third component, Shank-associated RH domain-interacting protein (SHARPIN). HOIP initiates an M1 linkage, and HOIL-1 adds an oxyester bond to form an M1/oxyester branched linkage (Rodriguez Carvajal et al., 2021).

Several single E3s were able to assemble multiple linkages, inducing mixed or branched chains (Fig. 2 C). For instance, Parkin assembles short-branched chains consisting of K6, K11, K48, and K63 linkages on mitochondrial outer membrane proteins (Fig. 2 C). These branched ubiquitin chains serve as tags for recruiting autophagic adapter proteins (Ordureau et al., 2020; Swatek et al., 2019). K29/K48 branched ubiquitin chains can be formed by HECT Domain E3 Ubiquitin Protein Ligase 1 (HECTD1) or Ubiquitin Protein Ligase E3C (UBE3C) (Chen et al., 2021; Harris et al., 2021; Leto et al., 2019). UBE3C-mediated branched ubiquitination regulates endoplasmic reticulum-associated protein degradation (ERAD). K6/K48 branched ubiquitin chains can be formed by an enterohemorrhagic *E. coli* (EHEC) effector protein called Non-LEE-encoded Ligase (NieL) (French et al., 2017; Hospenthal et al., 2013; Swatek et al., 2019; Valkevich et al., 2014). Except for UBE3C in ERAD and Parkin in mitophagy, these examples have been characterized only in vitro. Thus, the biological relevance of branched chains assembled by single E3s is an area for future studies.

Although cognate E3s are not known, there are certain E2s that prefer to branch off the chains and then elongate them. Yeast Ubc1 and the human ortholog UBE2K facilitate the formation of the K48/K63 branched ubiquitin chain (Pluska et al., 2021). The structural analysis showed that Ubc1 preferentially binds to K63-linked chain and modifies the proximal ubiquitin moiety with a K48 linkage (Pluska et al., 2021). Identification of the E3s that pair with these E2s will advance our understanding of the functions and mechanisms that regulate these branched chains.

### Deubiquitinases as branched ubiquitin chain editors

Disassembly of ubiquitin chains by DUBs limits the strength and duration of ubiquitin signals. DUBs recognize ubiquitin or ubiquitin chains for cleavage, and branching can modify this recognition (Table 1) (Harris et al., 2021; Kim et al., 2007; Oh et al., 2020; Wang et al., 2006).

Ubiquitin C-terminal hydrolase 37 (UCH37)/UCHL5 is a DUB that preferentially cleaves branched ubiquitin chains (Deol et al., 2020a; Song et al., 2021) (Fig. 2 D). It specifically debranches the K48 linkage from K6/K48, K11/K48, and K48/K63-branched chains. Structural analysis revealed that UCH37 has a binding site for a K48 linkage and an additional binding site for monomeric ubiquitin (Du et al., 2022). UCH37 associates with the proteasome through Rpn13 and promotes efficient degradation of substrates (Hamazaki et al., 2006; Qiu et al., 2006; Yao et al., 2006).

Besides UCH37, ubiquitin-specific peptidase 30 (USP30) is a K6 linkage-specific DUB (Cunningham et al., 2015) that can target K6/K48 branched linkages (Gersch et al., 2017). In in vitro assays, USP30 prefers K6/K48 branched linkage over any homotypic chain (Deol et al., 2020b). In addition, a comprehensive analysis of DUBs specific for K48/K63 branched linkages was recently reported, and the analysis identified MINDY3 and ATXN3 as debranching enzymes (Lange et al., 2023, *Preprint*). ATXN3 is known as a p97-interacting DUB and is involved in ER-associated degradation (ERAD) (Liu and Ye, 2012). ATXN3 is also known as a regulator of autophagy by deubiquitinating Beclin 1 (Ashkenazi et al., 2017). The biological relevance of the debranching activity of these DUBs is an interesting area for future studies. The biological relevance of the debranching activity of these DUBs is an interesting area for future studies.

Branching of ubiquitin chains can also restrict cleavage by DUBs. For example, the DUB A20 cleaves K63-linked chains from receptor interacting serine/threonine kinase 1 (RIPK1) to negatively regulate NF-κB signaling (Zinngrebe et al., 2014). The formation of M1/K63 mixed ubiquitin chains on RIPK1 inhibits this A20-dependent ubiquitin chain cleavage and promotes NF-κB signaling (Wertz et al., 2015). Similarly, branched K48/K63 chains protect K63-linkages from the DUB CYLD (Fig. 2 E). The cocrystal of CYLD and a K63-linked di-ubiquitin revealed that the K48 ubiquitin residue of the distal ubiquitin interacts with CYLD; thus, a K48 ubiquitin branch would likely interfere with this interaction (Ohtake et al., 2016).

Some DUBs are unaffected by branching. The TRAF-binding domain-containing protein (TRABID)/ZRANB1 is an ovarian tumor (OTU) family DUB that cleaves K29, K33, and to a lesser extent K63 linkages (Licchesi et al., 2011). A K29 linkage within K29/K48 branched chains conjugated on HECTD1 can be cleaved by TRABID and thereby protect HECTD1 from proteasomal degradation (Harris et al., 2021). Likewise, Cezanne cleaves K11 and oxyester linkages (Mevissen et al., 2016; Rodriguez Carvajal et al., 2021) and deubiquitinates K11/K48 linked chains assembled by APC/C, protecting APC/C substrates from proteasomal degradation (Bonacci et al., 2018; Cunningham et al., 2015; Deol et al., 2020b; Gersch et al., 2017; Lange et al., 2023, *Preprint*). Ubiquitin chain branching may affect their interaction with ubiquitin-binding domains (UBDs), generating unique coding signals. In this regard, branching can be viewed as a combinatorial modification of a ubiquitin moiety. In this crosstalk between two linkage types within a branched chain, a ubiquitin linkage can affect the assembly or disassembly of another linkage type. A comprehensive analysis of the interactions between branched linkages and UBDs would likely uncover new nodes and mechanisms of regulation.

### Non-lysine (K) ubiquitin linkages

Capturing S and T-linked ubiquitin chains may be technically more challenging due to the lower stability of an oxyester bond. Yet, among the six T residues within ubiquitin, four (T12, T14, T22, and T55) have been identified as ubiquitination sites initially from in vitro ubiquitination assays (Kelsall et al., 2019; Rodriguez Carvajal et al., 2021). Additionally, S20 in ubiquitin can be ubiquitinated by bacterial effector proteins in vitro (Bhogaraju et al., 2016).

Ubiquitination on S and T occurs via an ester bond, which is less stable than the isopeptide and peptide bonds found in lysine- and M1-linked ubiquitin chains (Kelsall, 2022). More recently, ubiquitin chains formed via T12, T14, and T22 as well as S20 were identified during the Toll-Like Receptor 7 (TLR7) signaling from cellular samples (McCrory et al., 2022). The discovery of S/T ubiquitination is in its infancy, and the function, dynamics, abundance, and stability of S/T-linked ubiquitin chains in cells under various conditions need to be further understood.

These unconventional ubiquitination sites were identified by mass spectrometry, revealing a peptide signature of two glycine residues (a derivative of the ubiquitin C-terminus) conjugated to a substrate peptide (Akimov et al., 2018; Ohtake et al., 2019; Olsen et al., 2006; Wagner et al., 2011). Although mass spectrometry can reveal novel conjugation types, capturing the dynamics of the stoichiometry and the length of the mixed or branched ubiquitin chains at an endogenous level remains a challenge. These non-canonical ubiquitin codes may provide a specific scaffold to unique “reader” proteins. To date, however, no ubiquitin decoder for S/T linkages is known in mammalian cells. This knowledge is required to elucidate the potential roles of S/T-linked ubiquitin chains.

**Figure 3.**
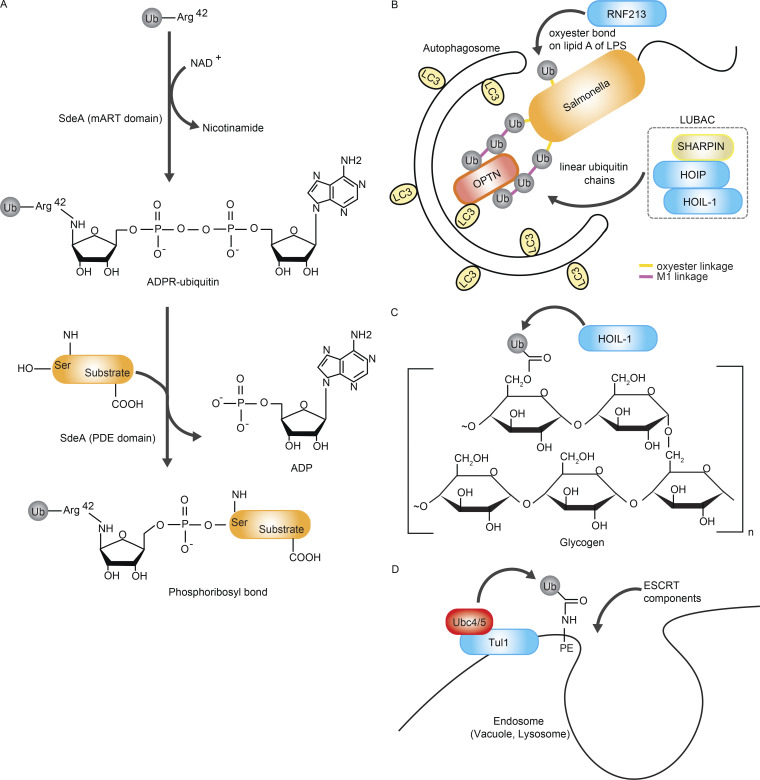
**Non-canonical ubiquitination and its biological relevance. (A)** The *Legionella pneumophila* effector SdeA facilitates S ubiquitination. Through its mono-ADP-ribosyltransferase (mART) activity, SdeA transfers ADP-ribose (ADPR) from NAD^+^ to Arginine(R) 42 of ubiquitin, forming ADPR–ubiquitin (Ub). Subsequently, the phosphodiesterase (PDE) domain of SdeA links ADPR–ubiquitin to the S residue on target proteins, resulting in the creation of ubiquitin-phosphoribosylated (PR) proteins. **(B)** The E3 ubiquitin ligase RNF213 plays a pivotal role in the ubiquitination of the lipid lipopolysaccharide (LPS), a component of the outer membrane in Gram-negative bacteria like *Salmonella*. RNF213-mediated ubiquitination of LPS is extended by LUBAC, resulting in the formation of linear ubiquitin chains. These ubiquitin chains act as recruitment signals for the protein OPTN, which bridges to LC3 on the autophagosome membrane, leading to the engulfment and degradation of *Salmonella* by autophagosomes. **(C)** The E3 ligase HOIL-1 can target the C6 hydroxy group of the sugar glycogen to form ubiquitinated glycogen. HOIL-1 may function as a quality control system that ubiquitinates inadequately branched glycogen to prevent the formation of polyglucosan bodies. **(D)** Ubiquitination of the lipid phosphatidylethanolamine (PE) is catalyzed by the yeast enzymes Ubc4/5 (E2) and Tul1 (E3). Ubiquitinated PE accumulates in endosomes, vacuoles, or lysosomes in cells, especially during starvation. Lysosomes containing ubiquitinated PE have been shown to recruit ESCRT components in vitro and may promote the formation of intraluminal vesicles of multivesicular bodies.

A few enzymes involved in S/T ubiquitination have been identified. The first group of enzymes consists of bacterial proteins responsible for S ubiquitination (Roberts et al., 2023; Tomaskovic et al., 2022). The SidE family of *Legionella* effector proteins, such as SdeA, catalyzes the formation of an ADP-ribose ubiquitin (ADPR ubiquitin) intermediate in the presence of NAD^+^ (Fig. 3 A) (Bhogaraju et al., 2016; Kalayil et al., 2018). SdeA then catalyzes the cleavage of the pyrophosphate bond in ADPR ubiquitin to form phosphoribosyl-ubiquitin, which conjugates to an S residue in the substrate, releasing ADP (Fig. 3 A) (Kalayil et al., 2018; Qiu et al., 2016). Phosphoribosyl-linked protein ubiquitination can be deconjugated by bacterial effectors SidJ, DupA, and DupB (Qiu et al., 2017; Shin et al., 2020). Furthermore, phosphoribosylation of ubiquitin impairs overall ubiquitination in host cells by preventing the activation of E1 and E2 enzymes of the conventional ubiquitination cascade (Bhogaraju et al., 2016). More recently, it was found that pR-ubiquitination by the Sde family is not limited to S-modification but also Tyr (Y) modification, providing a possibility of the diversity of ubiquitin modification by this family (Zhang et al., 2021).

The N-terminal end of proteins can be also targeted for ubiquitination. The ubiquitin-conjugating enzyme, UBE2W catalyzes ubiquitination on the N-terminal end of UCHL1 and UCHL5, altering their DUB activity (Davies et al., 2021). There are also several other proteins such as FKBP1A, SURF4, DUT, GADD45A, TXNL1, EIFR3G, TAM1, STAM, and PAPOLA targeted for the N-terminal ubiquitination in which the role of ubiquitination and the responsible enzymes remain unclear (Akimov et al., 2018).

The RING-type E3 ligase MYC Binding Protein 2 (MYCBP2) was identified as an unusual class of ubiquitin ligase through an activity-probe-based screen and catalyzes ubiquitination on T (Pao et al., 2018). Biochemical assays revealed that MYCBP2 represents a novel “RCR” E3 ligase, in which the RING domain interacts with the E2 ligase to facilitate the transfer of ubiquitin to its Tandem Cysteine (TC) domain, where the ubiquitin molecule is relayed through tandem cysteine residues and eventually to T on the substrate (Pao et al., 2018) (Fig. 1 B). A genome-edited mouse model expressing MYCBP2 with a catalytically inactive mutation exhibits neurodevelopmental phenotypes (Mabbitt et al., 2020; Virdee, 2022). The findings suggest that MYCBP2 functions in neurodevelopment and axon degeneration, but the mechanisms and substrates are unclear.

LUBAC mediates ubiquitin conjugation to the M1 residue of another ubiquitin molecule, forming chains via a peptide bond (Kirisako et al., 2006). These M1-linked ubiquitin chains are dependent on the LUBAC component HOIP and are important for controlling immune signaling cascades, such as the Tumor Necrosis Factor (TNF)-dependent NF-κB and apoptosis pathways. In contrast, HOIL-1 forms oxyester bonds between ubiquitin and S/T residues in its substrates (Kelsall et al., 2019). HOIL-1 can self-ubiquitinate and also targets IRAK1, IRAK2, and MyD88 in macrophages during Toll-like receptor signaling (Kelsall et al., 2019). Moreover, HOIL-1 conjugates a ubiquitin molecule to T12 and T55 of the M1-linked ubiquitin chains generated by HOIP (Rodriguez Carvajal et al., 2021). LUBAC that contains a catalytically inactive HOIL-1 mutant displays more efficient formation of M1-linked ubiquitin chains in vitro, suggesting that HOIL-1-mediated branching inhibits the elongation of M1-linked chains (Rodriguez Carvajal et al., 2021). These findings suggest that HOIL-1 might play distinct roles within and outside of LUBAC. Thus, elucidating the dynamics of LUBAC assembly under different conditions will be important to help disentangle HOIL-1 functions.

The Machado–Joseph disease (MJD) class DUBs were identified as non-lysine DUBs with highly specific ubiquitin esterase activity using an activity probe of ubiquitinated T (De Cesare et al., 2021). The cellular functions of different types of ubiquitin esterases are yet to be understood.

### Non-protein ubiquitin substrates: Sugars and lipids

Recent studies have shown that ubiquitination can occur on non-protein substrates (Ikeda, 2023; Sakamaki and Mizushima, 2023). The first non-protein substrate discovered was an outer component of Gram-negative bacteria, lipopolysaccharides (LPS). Ubiquitination of LPS is catalyzed by the RING-type ubiquitin ligase RNF213/Mysterin (Otten et al., 2021) (Fig. 3 B), a distinct E3 ligase linked to Moyamoya disease, a cerebrovascular disorder (Morito et al., 2014). Bacterial invasion of host cells induces a type of selective autophagy called xenophagy (Vargas et al., 2023). During xenophagy, bacteria become coated with ubiquitin, but the target substrate was long unknown since most studies focused on protein substrates. Unexpectedly, non-protein LPS ubiquitination was observed and found to restrict bacterial infection (Otten et al., 2021). Cryo-EM studies of RNF213/Mysterin revealed an N-terminal stalk, a dynein-like AAA-type ATPase domain, a RING domain, and E3 subdomains (Ahel et al., 2020). Strikingly, deletion of the RING domain did not affect RNF213 E3 ligase activity, suggesting an atypical mode of activity. This atypical E3 ligase activity depends on the RZ finger on the “E3 shell” of RNF213, distinct from its RING domain (Fig. 1 B) (Otten et al., 2021). Whether deubiquitinases exist that deconjugate ubiquitin from lipids remains a topic of ongoing investigation.

More recently, sugars (glycogen and maltoheptaose) were shown to be ubiquitinated by the RBR-type ubiquitin ligase HOIL-1 (Fig. 3 C) (Kelsall et al., 2022). Sugar ubiquitination by HOIL-1 with the E2 UbcH7 has been confirmed in vitro (Kelsall et al., 2022). Genetically modified mice expressing a HOIL-1 catalytically inactive mutant accumulated periodic acid-Schiff (PAS)-positive sugar aggregates in the brain, heart, lung, and liver, suggesting a link to HOIL-1-dependent sugar ubiquitination (Kelsall et al., 2022). The mechanism of substrate selection by HOIL-1 is entirely unknown and is key to further understanding its biological importance. It is possible that distinct HOIL-1 interactomes under specific cellular conditions influence the substrate selectivity of HOIL-1.

Phosphatidylethanolamine (PE), a phospholipid at endosomes and lysosomes, is the latest addition to the list of non-protein ubiquitin substrates (Sakamaki and Mizushima, 2023; Sakamaki et al., 2022). During autophagy, a “ubiquitin-like” protein LC3 conjugates to PE in forming autophagosomes. Whether ubiquitin also conjugates to PE, similar to LC3, was unknown. Mass spectrometry analysis of enriched lipid-containing fractions from yeast and mammalian cells identified a peptide signature of ubiquitin-conjugated PE (Fig. 3 D). In this case, ubiquitination occurs through amide bond formation. Ubiquitinated PE localized to endosomes and vacuoles/lysosomes, and the responsible enzymes were Uba1, Ubc4/5, and Tul1 in yeast (Sakamaki et al., 2022). Ubiquitination of PE occurs in cells and promotes recruitment of the endosomal sorting complex required for transport (ESCRT) (Sakamaki et al., 2022), but the precise molecular mechanisms and functions at the organismal level are yet to be revealed.

### Non-canonical ubiquitin codes in immune signaling pathways

**Figure 4.**
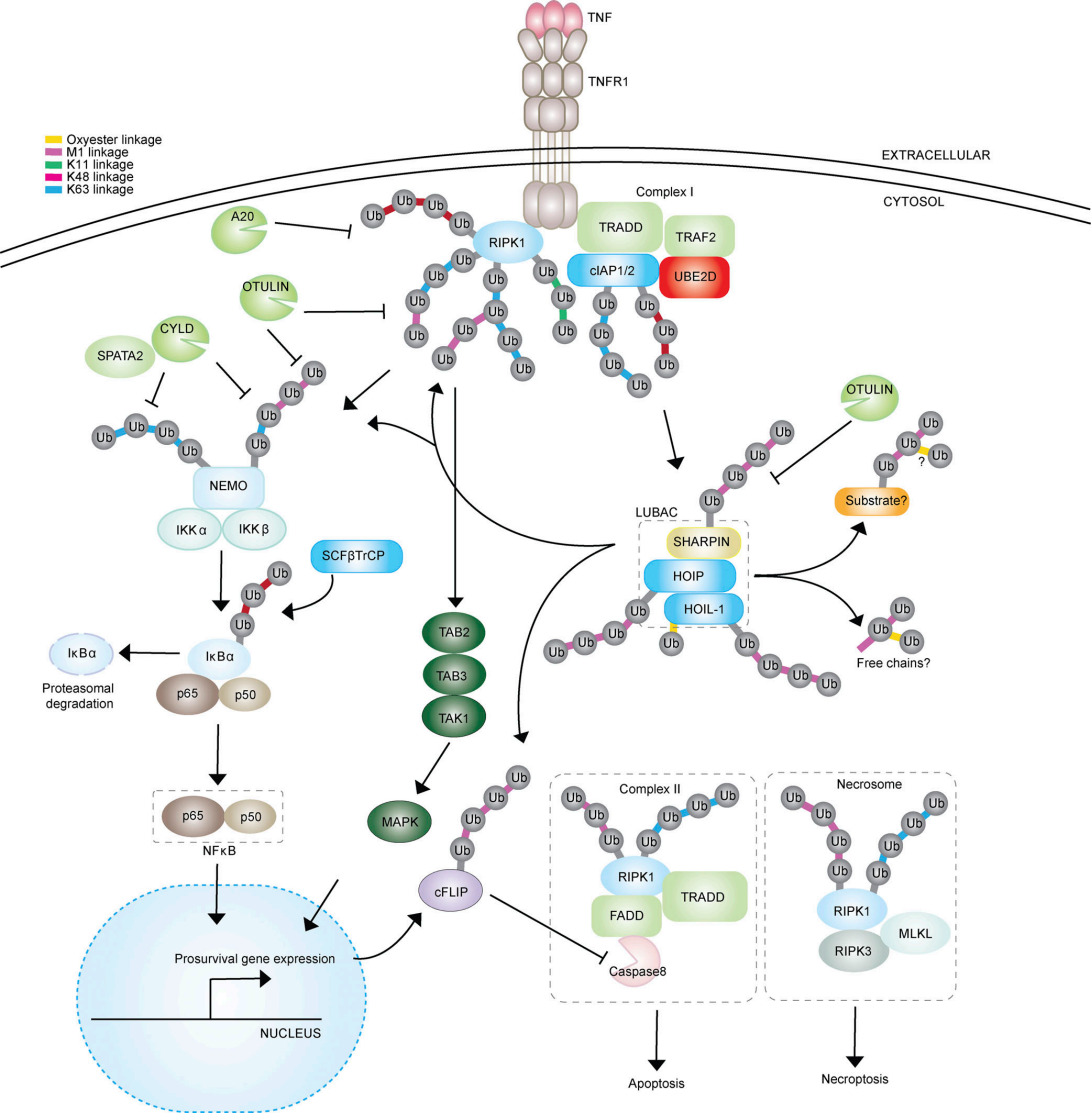
**The role of ubiquitination in TNF signaling.** TNF binding to TNFR1 triggers the formation of the TNFR complex I, which comprises TRAF2, TRADD, UBE2D, cIAP1/2, and RIPK1. Within TNFR complex I, RIPK1 undergoes ubiquitination with M1-, K11-, K48-, and K63-linked ubiquitin chains, including mixed and branched chains. This ubiquitination of RIPK1 serves to stabilize TNFR complex I and enables the transmission of downstream signaling through LUBAC-induced M1-linked ubiquitination of its constituents (HOIP, HOIL-1, and SHARPIN) and NEMO, resulting in the activation of the NF-κB (p65-p50) or MAPK signaling pathway to promote cell survival and proinflammatory gene expression. Extended TNF signaling triggers additional cytotoxic pathways: the apoptosis pathway mediated by the TNFR1 complex II, which includes RIPK1-FADD-Caspase8 and the necrosome-mediated pathway via the formation of the RIPK1–RIPK3–MLKL complex. Ubiquitinated substrates and free ester-linked branched chains catalyzed by LUBAC may play a central role in mediating inflammation and cell death signaling pathways.

The TNF-induced inflammatory pathway is regulated by various ubiquitin codes, including both canonical (K48-linked) and non-canonical (e.g., M1, K11, K29, K63-linked) linkages of diverse substrates (Fig. 4) (Asaoka and Ikeda, 2015; Cockram et al., 2021; Emmerich et al., 2016; Lork et al., 2017). TNF binding to the receptor TNFR triggers the formation of the TNFR complex I via recruitment of multiple factors such as the adaptor molecule TRADD, the ubiquitin ligases cIAPs, and the S/T kinase RIPK1 (Fig. 4). The initial phase is marked by self-ubiquitination of cIAPs with K63-linked and K48-linked chains, as well as ubiquitination of RIPK1 with different linkage types of chains (Huyghe et al., 2023; Varfolomeev and Vucic, 2022). Ubiquitination of these proteins leads to recruitment of other factors via their ubiquitin-binding domains, such as LUBAC components, I kappa B kinases (IKKs), and TGF-Beta Activated Kinase 1 (MAP3K7) Binding Protein 2 (TAB2)-TGF-Beta Activated Kinase 1 (TAK1) (Kanayama et al., 2004; Napetschnig and Wu, 2013; Rahighi et al., 2009). HOIL-1 catalyzes ubiquitin esterification, and its catalytic activity is critical in the TNF pathway (Fuseya et al., 2020), suggesting that ester-bond ubiquitin linkages are required to regulate this pathway. In the downstream cascade, phosphorylation of an inhibitory factor, IκB-α, leads to its K48-linked ubiquitination and proteasomal degradation, thus promoting nuclear translocation of NF-κB. RIPK1 is also ubiquitinated in the TNFR complex II, inducing apoptosis, and in the necrosome, leading to necroptosis (Huyghe et al., 2023). In the interleukin 1 (IL-1)-mediated signaling pathway, mixed chains of K11 and K63 linkages regulate NF-κB, which extended the concept of ubiquitin signaling tags beyond K63-homotypic chains (Ohtake et al., 2016). Overall, immune signaling pathways demonstrate the utility of complex ubiquitin codes for tight and sensitive spatiotemporal regulation.

### Perspective

The discovery of non-K48 ubiquitin codes first introduced the concept of ubiquitin functions beyond degradation. M1-linked ubiquitin chains, formed by the unique ubiquitin ligase complex LUBAC, revealed ubiquitination at non-lysine residues. Branched chains and their distinct architectures further diversify the ubiquitin code. Most recently, the identification of ubiquitination through oxy-ester bond formation has revealed non-protein substrates. Considering all these discoveries of unexpected ubiquitin linkage types, topologies, and substrates, our current understanding of “non-canonical ubiquitination” is likely far from complete. In this regard, new technologies to dissect ubiquitin architectures, such as the deubiquitinase-based molecular assay called Ub-clipping and top-notch mass spectrometry approaches (Deol et al., 2020a; Swatek et al., 2019) will likely reveal new types, functions, and mechanisms of non-canonical ubiquitination. For example, a high-resolution and high-precision mass spectrometry approach, parallel reaction monitoring (PRM)-based MS2 combined with Ub-clipping, revealed combinations of K48/K63 and K11/K48 branches induced by cIAP1 in cells (Akizuki et al., 2023). The stoichiometries of each branched linkage in cells are not extensively quantified, except for the abundance of K48/K63 branched linkages (Ohtake et al., 2016) and Parkin-generated branched linkages during mitophagy (Swatek et al., 2019). Since a substrate is often ubiquitinated at multiple sites in cells, new approaches to analyze a single ubiquitin chain on a single substrate are required to reveal the precise architecture of ubiquitin chains. Moreover, new reagents, such as the recently reported K48/K63 branched linkage-specific nanobody (Lange et al., 2023, *Preprint*), are needed to decipher the function of branched chains.

With several PROTACs entering clinical trials (Chirnomas et al., 2023), the field is poised for explosive growth. We envision new E3s that can be harnessed for neosubstrate degradation, the expansion of neosubstrate repertoires such as neurodegeneration-causing proteins, and strategies to improve the efficacy of target degradation. Importantly, the mechanisms of targeted protein degradation will need to be comprehensively understood. For example, since branched ubiquitin chains promote the degradation of neurodegeneration-causing aggregation-prone proteins in a proteasome-dependent manner (Yau et al., 2017), specific incorporation of branched ubiquitin chains may be beneficial for the targeted degradation of these neosubstrates. Moreover, intrinsic signaling pathways that enhance or inhibit targeted protein degradation are largely unknown. Identification of such cellular pathways may lead to better design of higher efficacy therapeutics using chemical degraders.

Future research will surely unveil new ubiquitin-mediated regulatory mechanisms that govern cellular functions, leading to the development of new strategies against human diseases.
